# Phosphoproteomic analysis of apoptotic hematopoietic stem cells from hemoglobin E/β-thalassemia

**DOI:** 10.1186/1479-5876-9-96

**Published:** 2011-06-25

**Authors:** Saranyoo Ponnikorn, Tasanee Panichakul, Kitima Sresanga, Chokdee Wongborisuth, Sittiruk Roytrakul, Suradej Hongeng, Sumalee Tungpradabkul

**Affiliations:** 1 Department of Biochemistry, Faculty of Science, Mahidol University, Bangkok, Thailand; 2 Faculty of Science and Technology, Suan Dusit Rajabhat University, Bangkok, Thailand; 3 Research Center, Faculty of Medicine Ramathobodi Hospital, Mahidol University, Bangkok, Thailand; 4 National Center for Genetic Engineering and Biotechnology, National Science and Technology Development Agency, Pathumthani, Thailand; 5 Department of Pediatrics, Faculty of Medicine Ramathibodi Hospital, Mahidol University, Bangkok. Thailand

**Keywords:** Phosphoproteome, Hemoglobin E/β-thalassemia, HSCs/CD34^+^, apoptosis

## Abstract

**Background:**

Hemoglobin E/β-thalassemia is particularly common in Southeast Asia and has variable symptoms ranging from mild to severe anemia. Previous investigations demonstrated the remarkable symptoms of β-thalassemia in terms of the acceleration of apoptotic cell death. Ineffective erythropoiesis has been studied in human hematopoietic stem cells, however the distinct apoptotic mechanism was unclear.

**Methods:**

The phosphoproteome of bone marrow HSCs/CD34^+ ^cells from HbE/β-thalassemic patients was analyzed using IMAC phosphoprotein isolation followed by LC-MS/MS detection. Decyder MS software was used to quantitate differentially expressed proteins in 3 patients and 2 normal donors. The differentially expressed proteins from HSCs/CD34^+ ^cells were compared with HbE/β-thalassemia and normal HSCs.

**Results:**

A significant change in abundance of 229 phosphoproteins was demonstrated. Importantly, the analysis of the candidate proteins revealed a high abundance of proteins that are commonly found in apoptotic cells including cytochrome C, caspase 6 and apoptosis inducing factors. Moreover, in the HSCs patients a significant increase was observed in a specific type of phosphoserine/threonine binding protein, which is known to act as an important signal mediator for the regulation of cell survival and apoptosis in HbE/β-thalassemia.

**Conclusions:**

Our study used a novel method to investigate proteins that influence a particular pathway in a given disease or physiological condition. Ultimately, phosphoproteome profiling in HbE/β-thalassemic stem cells is an effective method to further investigate the cell death mechanism of ineffective erythropoiesis in β-thalassemia. Our report provides a comprehensive phosphoproteome, an important resource for the study of ineffective erythropoiesis and developing therapies for HbE/β-thalassemia.

## Background

Beta-thalassemia (β-thalassemia) is an inherited disorder of hemoglobin (Hb) synthesis and is present in populations worldwide however, hemoglobin E/β-thalassemia (HbE/β-thalassemia) is particularly common in Southeast Asia [1]. In Thailand, 7% of the population carries the β-thalassemia trait and 17% carries the Hb E trait resulting in thirty-five thousand patients suffering from β-thalassemia syndrome [2]. HbE/β-thalassemia has variable symptoms including mild to severe anemia [3,4]. The excess of insoluble α chains accumulates in erythroid precursors forming inclusion bodies in the bone marrow as well as in the peripheral red blood cells. This leads to excessive intramedullary destruction of the erythroid precursors as previously described in β-thalassemia major [5]. Erythroid progenitor cells isolated from β-thalassemia major patients exhibited an ineffective erythropoiesis via apoptosis at the polychromatophilic normoblast stage [6] in either erythroid progenitor or erythroblast cells. It has been proposed that the precipitation of excess α-globin chains in marrow erythroid precursors as well as an excess of free iron could lead to oxidative stress and potentially to ineffective erythropoiesis [7,8]. However, the mechanism responsible for induction of apoptosis and the relevant signaling pathways in hematopoietic stem cells are still poorly understood. Hematopoietic stem cells (HSCs) contain specific markers on their surface such as CD34 and CD133 allowing for the isolation of relatively pure populations of these cells for *in vitro *study of the cellular processes in thalassemic stem cells. While previous attempts have focused on the cell culture system, in this study the phosphoproteome of the primary cells from the immediate collection of HSCs from patient blood or bone marrow was analyzed.

Mass spectrometry-based proteomics is an alternative method for the analysis of protein profiles and provides an extensive approach to analyze expressed proteins and discover potential biomarkers for diseases. The development of MS based technology has provided the opportunity to investigate cellular signaling mechanisms in terms of the phosphoproteome, the phosphopeptides or phosphoproteins present within a cell [9]. Protein phosphorylation is present on more than 30% of the proteome and regulates signal transduction pathways under normal conditions as well as in disorders such as diabetes, neurodegenerative diseases, autoimmune diseases and several forms of cancers [10]. Phosphorylation and dephosphorylation of specific amino acid residues, including serine, tyrosine and threonine, by specific kinases and phosphatases is a major form of post-translational modification in eukaryotic cellular machinery and can be regulated to occur at a particular time or in response to other stimuli. The levels of protein phosphorylation vary widely and specific sites may be phosphorylated from less than 1% to greater than 90% [11]. The global identification and characterization of phosphorylation is critical to the elucidation of signal transduction pathways, the understanding of the mechanism of disease progression, and the development of therapeutic applications [12]. The phosphoproteome of HSCs in HbE/β-thalassemic patients has not been previously investigated and analysis of changes in phosphorylation patterns may help in the understanding of the mechanisms that participate in apoptotic cell death leading to the ineffective erythopoiesis in bone marrow cells. Here, we described the first phosphoproteome analysis of HSCs from HbE/β-thalassemic patients using IMAC phosphoprotein isolation directly coupled with LC MS/MS analysis. Compared to healthy donors, HSCs from HbE/β-thalassemic bone marrow patients expressed high levels of several phosphoproteins, suggesting a role for these proteins in disease. These proteins were identified and categorized with regard to both the intrinsic and extrinsic pathway apoptotic pathways. Our results suggest that the 14-3-3 proteins might play a role in regulating apoptosis of HSCs in HbE/β-thalassemia.

## Experimental design and methods

### Samples of bone marrow

Bone marrow samples were obtained from three HbE/β-thalassemic patients admitted to Ramathibodi Hospital, Bangkok, Thailand and two normal donors. All patients were children 3-12 years-old and had symptoms of severe anemia and hepatosplenomegaly. Hemoglobin analysis showed no expression of the β-hemoglobin band with the Hb-E trait. Twenty milliliters of marrow was aspirated from the posterior iliac crest into syringes containing 0.1 ml of heparin and then shipped to the laboratory for stem cell isolation. Marrow collection was approved by the Ethical Committee of Research on Human beings at Ramathibodi Hospital, Faculty of Medicine, Mahidol University, Bangkok, Thailand and normal donors gave informed consent. All patients had not received blood transfusion within three months before they participated in the study.

### Isolation and culture of hematopoietic stem cells (HSCs)

Bone marrow samples (BM) from Hb E/β-thalassemic patients and healthy donors were collected for HSCs isolation. Briefly, mononuclear cells (MNCs) from BM were separated by using Isoprep™ solution (Lexis, Sweden). The MNCs were used to isolate the HSCs with a CD 34 isolation kit with magnetic microbead selection (Mini-MACS columns, Miltenyi Biotech, Germany). The method of isolation was performed as described in the manufacturer's protocol. 1-2 × 10^8 ^cells of MNCs were reacted with 100 μl of anti-CD 34 antibody coated with micro beads and 100 μl of anti-FcR antibody, at 4°C, for 30 min. After washing with PBS/2 mM EDTA/0.5%BSA buffer, cells were applied to a wet magnetic column. Next, buffer was added onto the column to remove non-binding cells. This step was repeated 4-5 times and CD 34^+ ^cells were then flushed from the column. The isolated HSCs/CD34^+ ^cells were harvested for phosphoproteomic analysis and cultivation of erythroid cells. The cultivation of erythroid cells derived from HSCs/CD34^+ ^cells was performed according to a procedure previously described [13] with some modifications. HSCs/CD34^+^cells were cultured in StemlineII medium (Sigma-Aldrich Corporation, Missouri, USA) supplemented with 100 ng/ml stem cell factor (SCF) (PeproTech, Rocky Hill, NJ, USA), 5 ng/ml IL-3 (R&D Systems, Inc., MN, USA), 10 μM hydrocortisone (Sigma-Aldrich), 100 μg/ml transferrin (Sigma-Aldrich), 100 μg/ml Humulin^®^N (Lilly PharmaFertigung UND Distribution Giessen, Germany), 0.18 μg/ml ferrous sulfate (Sigma-Aldrich), 0.16 mM monothioglycerol (Sigma-Aldrich), and 4 IU/ml erythropoietin (EPO; CilagAG International, Zug, Switzerland). All cultures were incubated at 37°C with 5% CO_2_. After culturing for 4 and 7 days, cells were collected for identification of erythroid markers and cell morphology. Cell surface membrane markers were analyzed to confirm cell types of HSCs and erythroid cells. Cell surface markers were detected with mouse anti-CD 34, 71, 45, and glycophorin A antibodies conjugated with fluorescent dye followed by analysis using flow cytometry (Beckman Coulter, USA). Cells were stained with Giemsa to visualize the morphology of erythroid cells.

### Phosphoprotein analysis

Phosphoproteins of HSCs/CD34^+ ^cells isolated from HbE/β-thalassemia patients and normal donors were analyzed in parallel by liquid chromatography in line with tandem MS mass spectrometry (Bruker, Germany). Briefly, freshly isolated CD34^+ ^cells were washed three times with phosphate-buffer saline (PBS), dissolved in lysis buffer containing 0.15 M NaCl, 5 mM EDTA, 1% Triton X100, 10 mM Tris-Cl, pH 7.4, 10 mM β-glycerophosphate, 25 mM NaF, 1 mM Na_3_VO_4_, 100 mM PMSF and complete protease inhibitor cocktail (Sigma-Aldrich), and then sonicated for 30 s. After centrifugation at 8,000 rpm for 15 min, the supernatant was collected and cell lysates were stored at -80°C until used. The phosphoproteins were enriched by immobilized metal affinity column (IMAC) (Pierce, Thermo Scientific, USA) according to the manufacturer's protocol. Phosphoproteins were reduced with 10 mM DTT, alkylated with 55 mM iodoacetamide, and digested with sequencing grade trysin (Promega, Germany) for 16 h at 37°C. Tryptic peptides were then concentrated by SpeedVac centrifugation and stored at -80°C prior to use. The iodoacetamide modified phosphopeptides were dissolved in 250 mM acetic acid/30% acetronitrile and protein concentration was determined by Lowry method [14]. Phosphopeptide samples were injected into a Ultimate 3000 LC System (Dionex, USA) coupled to ESI-Ion Trap MS (HCT Ultra PTM Discovery System, Bruker, Germany) with electrospray at a flow rate of 300 nl/min to a nanocolumn (Acclaim PepMap 100 C18, 3 μm, 100 A, 75 μm id × 150 mm). A Solvent gradient (Solvent A: H_2_O, 0.1% Formic acid; Solvent B: 20% H_2_O, 80% Acetronitrile, 0.1% Formic acid) was used with the following parameters: 10% - 70% B at 0-13 min, 90% B at 13-15 min and 10% B at 15-20 min. The resolution in MS step is 0.6 and the mass accuracy is 0.15 u (m/z).

### Proteins quantitation and identification

DeCyder MS Differential Analaysis software (GE Healthcare, USA) [15] was used for the differential quantitation of proteins and peptides based on MS signal intensities of individual LC-MS analyses. To evaluate the average abundance ratio of peptides from patients and normal donors quantitation of phosphopeptides, based on the peptide signal intensities, was performed using the Pepdetect module allowing for automated detection of peptides and assignment of charge states. Peptides were matched across different signal intensity maps between the patients and normal donors using the Pepmatch module. The relative abundances of peptides were expressed as ^2^log intensities with mass tolerance set to 0.5 amu and the retention time tolerance set to 2 min. All ^2^log intensities of patients were normalized with the ion intensity distribution of normal donors. An average abundance ratio > 2 fold was determined to be an over-expressed protein with a significant standard t-test *p-value *< 0.05.

The MS/MS data from DeCyderMS analysis was searched against the SwissProt database (SwissProt 05 516,603 sequences; 181,919,312 residues) for protein identification using Mascot software (Matrix Science, London, UK) [16]. Database interrogation was: taxonomy (Human or Eukaryote); enzyme (trypsin); variable modifications (carbamidomethyl, oxidation of methionine residues, phospho ST and phospho Y); mass values (monoisotopic); protein mass (unrestricted); peptide mass tolerance (2 Da); fragment mass tolerance (± 2 Da), peptide charge state (1+, 2+ and 3+) and three missed cleavages. Proteins considered as identified proteins had at least two peptides with an individual mascot score corresponding to *p < 0.05 *and *p < 0.1*, respectively. A mowse score of 20 or more was considered acceptable for proteins under MASCOT identification.

### Bioinformatic analysis

The phosphorylation sites of phosphopeptides from MS spectra were compared with the NetPHOS [17]http://www.cbs.dtu.dk/services/NetPhos/ phosphorylation site prediction for specific residues p-Tyr, p-Ser and p-Thr. The preferential phosphorylated residues were determined and the score with more than the threshold of all possible phosphorylation motifs was considered. The matching residues of selected peptides were reported and compared with the Phosphosite Plus database http://www.phosphositeplus.org to search for phosphorylation residues that have been previously investigated. Gene ontology and cellular pathway identification were analyzed using PANTHER http://www.pantherdb.org and UniprotKB http://www.uniprot.org databases.

## Results

**Figure 1:**
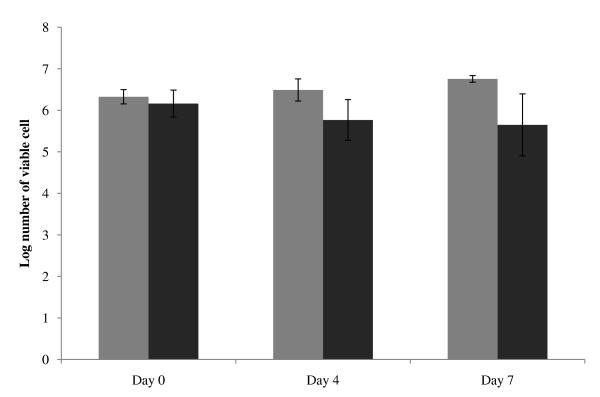
**Viability of HSCs/CD34^+ ^cells from HbE/β-thalassemic patients**. Cell numbers from 0, 4 and 7 day-old cultures were examined by trypan blue exclusion, numbers of viable cells from normal donors (grey) and HbE/β-thalassemic patients (black).

**Figure 2:**
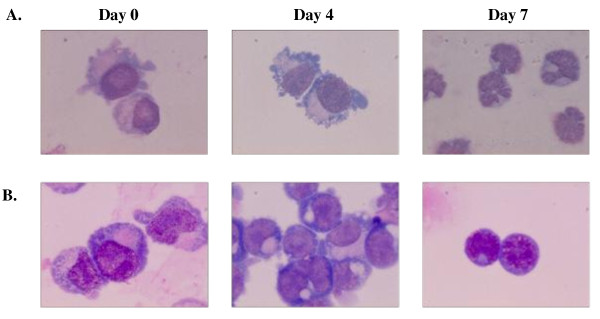
**Cultures of HSC/CD34^+ ^from HbE/β-thalassemic patients (A) and normal donors (B)**. After 7 days, the HbE/β thlassemic cell developed to erythroblasts and showed characteristic cell morphology of cells undergoing apoptosis including cell membrane blebbing and nuclear fragmentation.

HSCs/CD34^+ ^cells were isolated from 5 bone marrow samples belonging to 3 Hb E/β-thalassemic patients and 2 normal healthy donors. Flow cytometric analysis of confirmed that the isolated cells highly expressed the cell surface marker CD34 and had low levels of CD45 and were negative for glycophorin A. The purity of the isolated CD34^+ ^cells was 92% (data not shown). CD34^+ ^cells from patients and donors were cultured and cell viability was determined after 4 and 7 days of cultivation. The growth rate of thalassemic cells was reduced compared to normal cells (Figure 1). Giemsa staining revealed that CD34^+ ^cells from patients and donors had similar morphological characteristics to blast cells with a large nucleus and 2-3 nucleoli. The characteristics of apoptotic cells, including membrane blebbling and nuclear fragmentation, were only found in thalassemic cells at days 4 and 7 (Figure 2).

**Figure 3:**
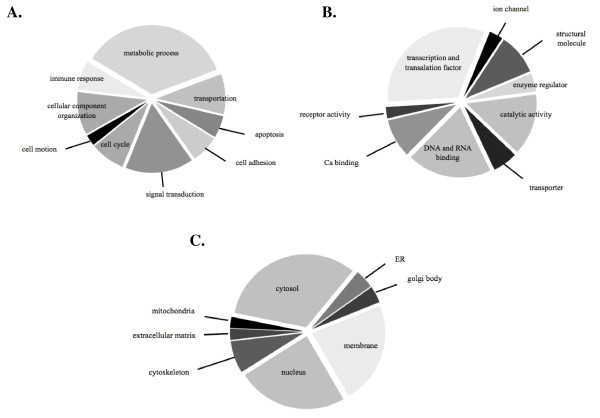
**Pie chart representing the characterization of identified phosphoproteins according to (A) biological processes, (B) molecular functions and (C) cellular localization**.

After LC-MS/MS analysis of the isolated phosphopeptides, all MS/MS spectra were searched against human protein database. A total of 347 peptides from CD34^+ ^cells were detected and found to correspond to 229 proteins which were encoded by 226 genes (additional file 1). Of the 347 unique peptide identifications, 204 phosphopeptides (58.79%) with 306 phosphorylation sites were identified. The specific amino acid residues of pSer:pThr:pTyr were represented as 67:31:2. The biological characterization of phosphoproteome in CD34^+ ^cells could be classified according to biological process, molecular function and cellular localization (Figure 3). The subcellular protein localization for identified phosphoproteins was available and included several cellular compartments. As expected in an investigation of the proteome, we identified an abundance of cytosol (33%), nucleus (24%) and membrane proteins (23%). The gene ontology analysis of our phosphoproteome revealed several proteins involved in basic molecular functions such as transcription/translation factors (32%), DNA/RNA binding (19%) and catalytic activity (14%). In addition to the biological characterization, the proteins could be identified specifically in metabolic processes (35%) and signal transduction (16%). Moreover, these proteins were categorized by PANTHER cellular pathway classification together with Uniprot gene ontology to match with 5 cellular pathways by various relationships such as protein interactions, modifications and regulation of expressions. However, this does not mean that all protein interactions are directly involved with a certain cellular pathway, but it enables identification of those biochemical pathways that are possibly altered in the HbE/β-thalassemic stem cell. In this study, five selected signaling pathways were represented in Table 1 with protein expression ratios between patients and normal donors greater than 2:1. These proteins were categorized as hematopoietic related proteins and other cellular pathway proteins (Table 1 and additional file 2).

**Table 1:** The selected relative abundance protein expression.

Gene Name	Description	Expression Ratio *
**Apoptosis-related protein**		
AIFM1_HUMAN	Apoptosis-inducing factor 1, mitochondrial	1.125
APC2_HUMAN	Adenomatous polyposis coli protein 2	2.315
CASP6_HUMAN	Caspase 6	5.559
CTBL1_HUMAN	Beta-catenin-like protein 1	1.127
CYC_HUMAN	Cytochrome c	9.480
FOXA1_HUMAN	Hepatocyte nuclear factor 3-alpha	4.021
LMNA_HUMAN	Lamin-A/C	1.120
MAP2_HUMAN	Microtubule-associated protein 2	1.07
PRKDC_HUMAN	DNA-dependent protein kinase catalytic subunit	3.423
RN5A_HUMAN	2-5A-dependent ribonuclease	1.41
SGPL1_HUMAN	Sphingosine-1-phosphate lyase 1	2.305
ST17A_HUMAN	Serine/threonine-protein kinase 17A	1.137
TNFL6_HUMAN	Tumor necrosis factor ligand superfamily member 6	0.9352
TNR12_HUMAN	Tumor necrosis factor receptor superfamily member 12A	1.094
TAU_HUMAN	Microtubule-associated protein tau	5.023
**p53 signaling pathway**		
1433S_HUMAN	14-3-3 protein sigma	2.274
1433Z_HUMAN	14-3-3 protein zeta/delta	2.907
1433G_HUMAN	14-3-3 protein gamma	2.299
BC11B_HUMAN	B-cell lymphoma/leukemia 11B	1.125
SHSA5_HUMAN	Protein shisa-5	1.143
CTBL1_HUMAN	Beta-catenin-like protein 1	1.172
**Ubiquitin proteasome pathway**		
HUWE1_HUMAN	E3 ubiquitin HUWE1	3.477
SMUF1_HUMAN	E3 ubiquitin SMUF1	1.099
CBL_HUMAN	E3 ubiquitin CBL	5.746
DCA15_HUMAN	DDB1	2.290
OTU7A_HUMAN	OTU domain	1.071
HERC3_HUMAN	Probable E3 ubiquitin HERC3	4.907
UBQL1_HUMAN	Ubiquilin	1.275
PSA7_HUMAN	Proteasome subunit alpha type	1.161
**WNT signaling pathway**		
APC2_HUMAN	Adenomatous polyposis coli protein 2	2.315
PCDH7_HUMAN	Protocadherin-7	2.252
HXA7_HUMAN	Homeobox protein Hox-A7	1.602
WNT8B_HUMAN	Protein Wnt-8b	1.094
LRP5_HUMAN	Low-density lipoprotein receptor-related protein 5	1.263
**PI3 Kinase signaling pathway**		
FOXF2_HUMAN	Forkhead box protein F2	2.273
FOXA1_HUMAN	Hepatocyte nuclear factor 3-alpha	4.021
1433S_HUMAN	14-3-3 protein sigma	2.274
1433Z_HUMAN	14-3-3 protein zeta/delta	2.907
1433G_HUMAN	14-3-3 protein gamma	2.299

*: Decyder MS analysis at significant t-test *p < 0.05 *was performed the peptide intensity. The relative expression ratio was derived by the peptide ion intensity of patient normalized with normal donor.

**Figure 4:**
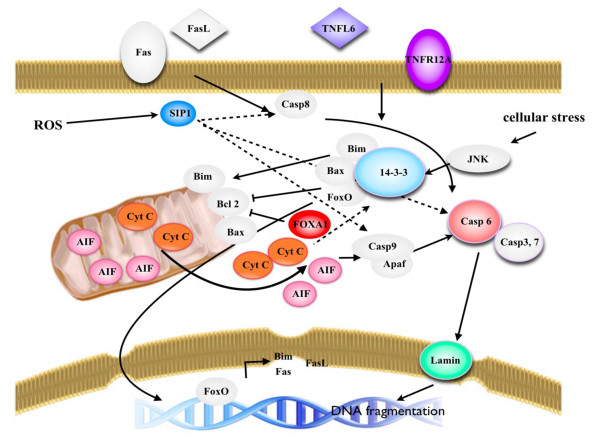
**Schematic diagram of candidate phosphoproteins implicated in apoptotic pathways**. The color pictures represent proteins that were identified in this study, the grey pictures are proteins that have been previously identified.

Interestingly, Several proteins that were over-expressed in patients were identified as apoptotic proteins. Of these, five proteins were characterized as common in the apoptotic pathway including cytochrome C, apoptosis inducing factor 1 (AIFM1), caspase 6, tumor necrosis factor ligand 6 (TNFL 6) and tumor necrosis factor receptor super family 12A (TNFR 12A or TWEAK). These proteins were involved in both mitochondrial dependent apoptosis (intrinsic mechanism) and death receptor mediated apoptosis (extrinsic mechanism). Previous evidence suggests that the pathology and disease progression of β-thalassemia relates to the accumulation of reactive oxygen species (ROS), which can generate DNA adducts in the nucleus and induce the DNA damage pathway. In this study, six proteins encoded by four genes were identified and found to be responsible for DNA damage and responsive mechanisms such as the p53 pathway. Interestingly, we found the isoforms sigma, zeta/delta and gamma of the 14-3-3 protein were over-expressed in patients more than 2 fold as compared with normal donors. These 14-3-3 proteins are involved in cellular pathways including apoptosis and phosphatidyl inositol 3 kinase activity by PANTHER pathway analysis. A schematic diagram of the apoptotic pathway including the identified phosphoproteins in HbE/β-thalassemic stem cells was proposed (Figure 4).

## Discussion

Conventional methods typically have difficulty using fresh human hematopoietic stem cells for studying cell signaling in the apoptotic pathway because of the limited amount of sample from patients and normal donors. On the other hand, *in vitro *culture is capable of sustaining a large number of cells, which can confer specific advantages and disadvantages. The use of freshly isolated human hematopoietic stem cells from thalassemic patients and normal donors to specifically investigate apoptotic mechanisms by avoiding the culture system was achieved in this study using phosphoproteomic technology. The IMAC technique indicated more than 50% phosphopeptide specificity, which is similar to that previously described [18]. Likewise, several studies have reported that direct phosphoproteins/peptides enrichment with TiO_2 _could improve the sensitivity and efficacy of phosphoproteome study [18,19]. Decyder MS is an alternative approach to quantitative proteomics based on LC-MS/MS analysis of peptides without incorporating an isotopic label. The matching of peptides across patients and normal donors requires accurate mass and reproducible retention times [15] and some quantitative proteomic techniques have been proposed [20].

Ineffective erythropoiesis is one important complication in thalassemic disease that contributes to the abnormal erythroid cell expansion and differentiation in bone marrow [21,22]. Apoptotic cell death has been described as the major contributor as seen in β-thalassemic bone marrow [5] and even in peripheral blood stem cells *in vitro *[23]. The bone marrow of patients with β-thalassemia contains five to six times the number of erythroid precursors as the healthy controls [24]. The acceleration of apoptosis and ineffective erythropoiesis in erythroid precursor cells and HSCs/CD34^+ ^has been observed with apoptotic cells present at 15 times greater levels in β-thalassemic patients compared to normal donors [5,7]. The acceleration of apoptosis has been characterized in erythroid cells at the polychromatophilic and orthochromic nomoblast stages during erythroid differentiation [6]. However, using a thalassemic mouse model of β-thalassemia major and thalassemia intermedia, it was suggested that apoptosis and hemolysis were not the major causes for the ineffective erythropoiesis. Results suggested that an increase in erythroid cell proliferation but not cell differentiation contributed to the ineffective erythopoiesis [25]. However, it remains unclear whether the expansion of erythroid cells but not cell differentiation can directly or indirectly contribute to the acceleration of apoptosis in β-thalassemia. Our study in HbE/β-thalassemic HSCs has shown that the growth reduction of erythroid cells derived from HbE/β-thalassemic HSCs was substantially due to induction of apoptosis. This was supported by the observation of characteristic apoptotic cell morphology and phosphoproteomic analysis of HbE/β-thalassemia. Our phosphoproteome data has identified specific apoptotic related proteins from HSCs/CD34^+ ^cells in HbE/β-thalassemia. We categorized candidate proteins into two major groups. First is the common apoptotic proteins associated with the extrinsic and intrinsic cell death pathway. Another is a non-distinct mechanism in both cell death pathways that has been addressed in several studies. Cytochrome C and apoptosis inducing factor 1 (AIFM1) are the main common components of the intrinsic and the mitochondria dependent apoptotic pathway. The activation of various death stimuli triggers the localization of pro-apoptotic proteins Bcl-2 and BH3 family into mitochondria [26,27]. Consequently, the release of cytochrome C mediates apoptosome formation and effective caspase cascade activation. Additionally, AIFM1 is activated by the same response that elicits chromatin condensation in the nucleus [28-30]. In addition, a death receptor mediated pathway seems to be implicated in apoptosis during erythropoiesis with Fas-Fas ligand interactions [31,32]. We have identified additional factors in the death receptor mediated pathway including the tumor necrosis factor ligand member 6 (TNFL 6), tumor necrosis factor receptor superfamily member 12A (TNFRST 12A) and caspase 6. Both TNFL 6 and TNFRSF 12A are involved in FAS mediated apoptosis or death receptor mediated apoptosis [33,34], while caspase 6 is generally considered as an effective caspase that is cleaved by caspase-3 after the activation of the caspase cascade [35]. Caspase 6 has an important role in the regulation of chromatin condensation through the cleavage of nuclear laminar [36]. Interestingly, we have also identified lamin A or nuclear lamin A (LMNA), which is a component of nuclear laminar proteins, in our analysis. A second group of apoptotic related proteins was also over-expressed in patients. For example, FOXA1 (hepatocyte nuclear factor alpha) is a transcription factor that is involved in embryonic development and regulation of gene expression in differentiated tissues [37]. FOXA1 is involved in regulating apoptosis by inhibiting expression of the anti-apoptotic protein Bcl-2 [38]. Another protein highly expressed is sphingosine 1 phosphate layase 1 (S1P), one type of sphingosine metabolite. S1P plays an important role in regulation of cell proliferation, survival and death [39] and is generated during lipid peroxidation by reactive oxygen species (ROS) accumulation inside the cell [40]. Increasing of S1P or endogenous sphingosine levels triggers multiple mechanisms to induce apoptosis through the activation of caspases [40]. The accumulation of ROS is a common complication in β-thalassemia. ROS was reported to induce lipid peroxidation that contributes to DNA damage [41,42].

Nevertheless, *in vitro *studies in erythroid cells have demonstrated that particular pathways are involved directly or indirectly in apoptosis and ineffective erythropoiesis in HbE/β-thalassemia [23,43]. However, the upstream signaling events in the progression of β-thalassemia have not been identified. Our investigation of the phosphoproteome was performed using a novel procedure with freshly isolated HSCs/CD34^+^cells, allowing for analysis of cell signaling. The related cellular pathways could be identified and their possible roles in the pathogenesis of β-thalassemia could be examined. Phosphoproteins are known to mediate various pathways and interestingly, we identified 14-3-3 isoforms sigma, gamma and zeta/delta that are linked to three cellular pathways including p53, phosphatidyl inositol 3 (PI3) kinase, and apoptosis. The 14-3-3 proteins are a family of multifunctional phosphoserine/phosphothreonine binding molecules and are involved in various cellular processes including cell survival, cell cycle progression and apoptosis [44,45]. Ultimately, the regulation of 14-3-3 function is controlled by various upstream signal transduction pathways in particular conditions such as DNA damage and oxidative stress promote phosphoprylation/dephosphorylation of specific site [46,47]. In the case of apoptosis, the cytosolic 14-3-3 protein down regulates the p53 pathway and the JNK signaling pathway [44,45] but mediates the mitochondrial dependent apoptotic pathway. JNK promotes the pro-apoptotic protein, Bax, which is translocated to the mitochondria through phosphorylation of 14-3-3, a cytoplasmic anchor of Bax. Phosphorylation of 14-3-3 leads to dissociation of Bax from this protein and induces the mitochondrial dependent apoptotic pathway through cytochrome C release followed by effective caspase activation [48]. Besides the pro-apoptotic proteins, the 14-3-3 protein also regulates the localization of other apoptotic signaling proteins such as c-Abl. In response to DNA damage, JNK induces phosphorylation of the 14-3-3 protein and releases the binding of c-Abl from cytosol to the nucleus [49]. In the case of hematopoietic stem cell homeostasis, 14-3-3 responds to various physiological stimuli that can contribute to cell survival as well as death. The 14-3-3 protein binds to the FoxO protein, one member of the forkhead transcription factors. Specifically, FoxO3 and FoxO4 are down regulated by 14-3-3, which retains FoxO in the cytosol under normal physiological conditions. Upon DNA damage and oxidative stress, JNK can phosphorylate the 14-3-3 protein and leads to the dissociation of both FoxO proteins from their partner [48,50]. Consequently, FoxO3 can localize to the nucleus and regulate expression of Bim and FasL, which play an important role in apoptotic cell death in HSCs [51-54]. The 14-3-3 protein was also found in stem cell disease using the proteomics approach [55]. The investigation of bone marrow and peripheral blood blast cells in acute amyloid leukemia (AML) by 2-DE revealed the expression of 14-3-3 related proteins. Moreover, another study in leukemic stem cells identified the 14-3-3 phosphorylation site motif in candidate phosphopeptides. The results from SCANSITE analysis indicate that most phosphopeptides are phosphorylated by a kinase 14-3-3 module. Therefore, it was proposed that 14-3-3 may regulate the oncogenic pathway in leukemic stem cell disease [19]. A recent study of 14-3-3 protein-protein interaction by quantitative mass spectrometry in multiple myeloma revealed novel 14-3-3 zeta interacting proteins that may have various biological functions and may regulate the activity of apoptotic proteins such as Bax, cytochrome C and caspase 6 [56]. Therefore, 14-3-3 zeta is likely to play an important role in apoptosis.

## Conclusions

We have observed the over-expression of the 14-3-3 protein isoforms sigma, gamma and zeta/delta in HbE/β-thalassemic stem cells. The 14-3-3 protein may be a critical mediator of the signaling pathway that regulates between cell survival and death due to ineffective erythropoiesis in β-thalassemic patients. Finally, this study has shown that a significant amount of common apoptotic proteins are found in the patients with HbE/β-thalassemia and these proteins correspond to the disease complications in apoptosis of HSCs in patients. These experiments used a novel method to understand the proteins that influence a particular pathway in a given disease or physiological condition. Ultimately, our results demonstrate that phosphoproteome profiling in HbE/β-thalassemic stem cells is an effective technique to investigate the cell death mechanism of ineffective erythropoiesis in β-thalassemia.

## Abbreviations

HSCs/CD34^+^: CD34^+ ^hematopoietic stem cells; HbE/β-thalassemia: hemoglobin E beta thalassemia; IMAC: immobilized metal affinity column; LC-MS/MS: liquid chromatography with tandem mass spectrometry; PANTHER: Protein analysis through evolutionary relationships.

## Competing interests

The authors declare that they have no competing interests.

## Authors' contributions

SP - performed experiments, interpreted results and drafted manuscript, TP - performed experiments, interpreted results and drafted manuscript, KS - performed bioinformatic analysis, CW - performed flow cytometry analysis, SR - designed and interpreted MS data, SH - designed, collected and interpreted clinical data, ST- designed and interpreted experiment, prepared final manuscript. All authors read and approved the final manuscript.

## Supplementary Material

Additional file 1 **Table containing the 229 proteins with MS identified phosphorylation site and the comparison of phosphorylation site using bioinformatic analysis**. Click here for file

Additional file 2 **Table indicating the cellular pathway analysis of identified proteins in this study**. Click here for file
