# Supplementary material for: Phosphoproteomic analysis of apoptotic hematopoietic stem cells from hemoglobin E/β-thalassemia
Source: J Transl Med. 2011 Jun 25;9:96. doi: 10.1186/1479-5876-9-96 (PMC3142509; doi:10.1186/1479-5876-9-96)
Supplement: Additional file 2 — Table indicating the cellular pathway analysis of identified proteins in this study. [file 1479-5876-9-96-S2.PDF]

APOPTOSIS		
Uniprot	expression ratio	NAME
ADA11_HUMAN	3.43	Disintegrin and metalloproteinase domain-containing protein 11
AIFM1_HUMAN	1.125	Apoptosis-inducing factor 1, mitochondrial
APC2_HUMAN	2.315	Adenomatous polyposis coli protein 2
CASP6_HUMAN	5.559	Caspase 6
CNTN1_HUMAN	1.265	Contactin 1
CNTN4_HUMAN	1.172	Contactin 4
CTBL1_HUMAN	1.127	Beta-catenin-like protein 1
CYC_HUMAN	9.48	Cytochrome c
DDR1_HUMAN	2.281	Epithelial discoidin domain-containing receptor 1
FOXA1_HUMAN	4.021	Hepatocyte nuclear factor 3-alpha
IFT57_HUMAN	1.085	Intraflagellar transport protein 57 homolog
LMNA_HUMAN	1.12	Lamin-A/C
MAP2_HUMAN	1.07	Microtubule-associated protein 2
MKL1_HUMAN	1.25	MKL/myocardin-like protein 1
NLRC3_HUMAN	1.081	Protein NLRC3
PRKDC_HUMAN	3.423	DNA-dependent protein kinase catalytic subunit
RALB_HUMAN	2.168	Ras-related protein Ral-B
RN5A_HUMAN	1.41	2-5A-dependent ribonuclease
SGPL1_HUMAN	2.305	Sphingosine-1-phosphate lyase 1
SHSA5_HUMAN	1.143	Protein shisa-5
ST17A_HUMAN	1.137	Serine/threonine-protein kinase 17A
TNFL6_HUMAN	0.9352	Tumor necrosis factor ligand superfamily member 6
TNR12_HUMAN	1.094	Tumor necrosis factor receptor superfamily member 12A
UBQL1_HUMAN	1.275	Ubiquilin-1
ULK1_HUMAN	1.098	Serine/threonine-protein kinase ULK1
TAU_HUMAN	5.023	Microtubule-associated protein tau
1433S_HUMAN	2.274	14-3-3 protein sigma
1433Z_HUMAN	2.907	14-3-3 protein zeta/delta
1433G_HUMAN	2.299	14-3-3 protein gamma
**p53 signaling pathway**		
1433S_HUMAN	2.274	14-3-3 protein sigma
1433Z_HUMAN	2.907	14-3-3 protein zeta/delta
1433G_HUMAN	2.299	14-3-3 protein gamma
BC11B_HUMAN	1.125	B-cell lymphoma/leukemia 11B
SHSA5_HUMAN	1.143	Protein shisa-5
CTBL1_HUMAN	1.172	Beta-catenin-like protein 1
**Ubiquitin proteasome pathway**		
HUWE1_HUMAN	3.477	E3 ubiquitin-protein ligase HUWE1
SMUF1_HUMAN	1.099	E3 ubiquitin-protein ligase SMURF1
CBL_HUMAN	5.746	E3 ubiquitin-protein ligase CBL
DCA15_HUMAN	2.29	DDB1- and CUL4-associated factor 15
OTU7A_HUMAN	1.071	OTU domain-containing protein 7A
HERC3_HUMAN	4.907	Probable E3 ubiquitin-protein ligase HERC3
UBQL1_HUMAN	1.275	Ubiquilin-1
PSA7_HUMAN	1.161	Proteasome subunit alpha type-7

**WNT signaling pathway**		
APC2_HUMAN	2.315	Adenomatous polyposis coli protein 2
PCDH7_HUMAN	2.252	Protocadherin-7
HXA7_HUMAN	1.602	Homeobox protein Hox-A7
WNT8B_HUMAN	1.094	Protein Wnt-8b
LRP5_HUMAN	1.263	Low-density lipoprotein receptor-related protein 5
**HUNTINGTON disease**		
GRIK3_HUMAN	1.143	Glutamate receptor, ionotropic kainate 3
TAF4_HUMAN	1.133	Transcription initiation factor TFIID subunit 4
IFT57_HUMAN	1.085	Intraflagellar transport protein 57 homolog
DC1L2_HUMAN	2.243	Cytoplasmic dynein 1 light intermediate chain 2
SP8_HUMAN	1.169	Transcription factor Sp8
CASP6_HUMAN	5.559	Caspase-6
**Parkinson Disease**		
1433S_HUMAN	2.274	14-3-3 protein sigma
1433Z_HUMAN	2.907	14-3-3 protein zeta/delta
1433G_HUMAN	2.299	14-3-3 protein gamma
PSA7_HUMAN	1.161	Proteasome subunit alpha type-7
**PI3 Kinase signaling pathway**		
FOXF2_HUMAN	2.273	Forkhead box protein F2
FOXA1_HUMAN	4.021	Hepatocyte nuclear factor 3-alpha
1433S_HUMAN	2.274	14-3-3 protein sigma
1433Z_HUMAN	2.907	14-3-3 protein zeta/delta
1433G_HUMAN	2.299	14-3-3 protein gamma
**TGF-β signaling pathway**		
FOXF2_HUMAN	2.273	Forkhead box protein F2
FOXA1_HUMAN	4.021	Hepatocyte nuclear factor 3-alpha
SMUF1_HUMAN	1.099	E3 ubiquitin-protein ligase SMURF1
INHBC_HUMAN	2.322	Inhibin beta C chain
**EGF receptor signaling pathway**		
1433S_HUMAN	2.274	14-3-3 protein sigma
1433Z_HUMAN	2.907	14-3-3 protein zeta/delta
1433G_HUMAN	2.299	14-3-3 protein gamma
CBL_HUMAN	5.746	E3 ubiquitin-protein ligase CBL
PHLB2_HUMAN	2.151	Pleckstrin homology-like domain family B member 2
**FGF signaling**		
1433S_HUMAN	2.274	14-3-3 protein sigma
1433Z_HUMAN	2.907	14-3-3 protein zeta/delta
1433G_HUMAN	2.299	14-3-3 protein gamma
**Hematopoiesis related protein**		
CBL_HUMAN	5.746	E3 ubiquitin-protein ligase CBL
CLC4A_HUMAN	3.888	C-type lectin domain family 4 member A
HIF1A_HUMAN	5.914	Hypoxia-inducible factor 1-alpha
CDCP1_HUMAN	6.24	CUB domain-containing protein 1
MEFV_HUMAN	1.154	Pyrin
HUWE1_HUMAN	3.477	E3 ubiquitin-protein ligase HUWE1

PSA7_HUMAN	1.161	Proteasome subunit alpha type-7
SDCG8_HUMAN	2.202	Serologically defined colon cancer antigen 8
RCL1_HUMAN	3.448	RNA 3'-terminal phosphate cyclase-like protein
